# Gene expression analysis of skin grafts and cultured keratinocytes using synthetic RNA normalization reveals insights into differentiation and growth control

**DOI:** 10.1186/s12864-015-1671-5

**Published:** 2015-06-25

**Authors:** Shintaro Katayama, Tiina Skoog, Eeva-Mari Jouhilahti, H. Annika Siitonen, Kristo Nuutila, Mari H Tervaniemi, Jyrki Vuola, Anna Johnsson, Peter Lönnerberg, Sten Linnarsson, Outi Elomaa, Esko Kankuri, Juha Kere

**Affiliations:** Department of Biosciences and Nutrition, Karolinska Institute and Center for Innovative Medicine, Huddinge, Sweden; Folkhälsan Institute of Genetics, Helsinki, Finland; Department of Medical Genetics, Haartman Institute and Research Programs Unit, Molecular Neurology, University of Helsinki, Helsinki, Finland; Department of Pharmacology, Faculty of Medicine, University of Helsinki, Helsinki, Finland; Helsinki Burn Center, Helsinki University Central Hospital, University of Helsinki, Helsinki, Finland; Department of Medical Biochemistry and Biophysics, Karolinska Institutet, Stockholm, Sweden; Science for Life Laboratory, Solna, Sweden

## Abstract

**Background:**

Keratinocytes (KCs) are the most frequent cells in the epidermis, and they are often isolated and cultured *in vitro* to study the molecular biology of the skin. Cultured primary cells and various immortalized cells have been frequently used as skin models but their comparability to intact skin has been questioned. Moreover, when analyzing KC transcriptomes, fluctuation of polyA+ RNA content during the KCs’ lifecycle has been omitted.

**Results:**

We performed STRT RNA sequencing on 10 ng samples of total RNA from three different sample types: i) epidermal tissue (split-thickness skin grafts), ii) cultured primary KCs, and iii) HaCaT cell line. We observed significant variation in cellular polyA+ RNA content between tissue and cell culture samples of KCs. The use of synthetic RNAs and SAMstrt in normalization enabled comparison of gene expression levels in the highly heterogenous samples and facilitated discovery of differences between the tissue samples and cultured cells. The transcriptome analysis sensitively revealed genes involved in KC differentiation in skin grafts and cell cycle regulation related genes in cultured KCs and emphasized the fluctuation of transcription factors and non-coding RNAs associated to sample types.

**Conclusions:**

The epidermal keratinocytes derived from tissue and cell culture samples showed highly different polyA+ RNA contents. The use of SAMstrt and synthetic RNA based normalization allowed the comparison between tissue and cell culture samples and thus proved to be valuable tools for RNA-seq analysis with translational approach. Transciptomics revealed clear difference both between tissue and cell culture samples and between primary KCs and immortalized HaCaT cells.

**Electronic supplementary material:**

The online version of this article (doi:10.1186/s12864-015-1671-5) contains supplementary material, which is available to authorized users.

## Background

Skin is a multi-layered tissue that is composed of continuously renewing epidermis – with keratinocytes (KCs) as a predominant cell type – and underlying dermis populated mostly by fibroblasts. The life span of epidermal keratinocytes is controlled by two alternative pathways: differentiation as their normal function or activation as an altered function in wound healing or skin diseases [1]. Epidermal KCs residing in the basal layer of the epidermis differentiate through multiple layers and finally shed as cornified dead cells from the skin surface [2, 3].

The relatively noninvasive sampling together with the methods that allow culturing of pure KCs have greatly facilitated research on skin and KCs. In cell culture, KCs are uncoupled from their tissue environment that naturally provides a network of homeostatic control signals; they are induced to either retain an active proliferative state or to differentiate. However, the prolonged KC culturing leads to the induction of cellular senescence [4] and therefore not only primary KCs but also immortalized KC lines, such as HaCaT (a spontaneously immortalized cell line) [5], have been widely studied to understand various normal and altered functions of the skin. HaCaT cells represent a highly popular model system since despite some UV-inducible mutations in *TP53* alleles [6, 7] they are non-tumorigenic and have retained their capacity to differentiate [5, 7, 8]. The comparability of each of the models to intact skin has often been questioned. In the current study, we address the question of how representative models cultured KCs and HaCaTs are for studying human epidermis.

Genome-wide expression profiling is an useful approach to screen key genes with respect to different cellular statuses and to further model the regulatory networks [9]. Microarray technology provides a traditional profiling method to measure thousands of known genes simultaneously, but it has recently been replaced by RNA-seq technology that has proven to give more detailed insights into transcriptome. Both technologies have been previously applied to study the gene expression in skin [10–12]. However, an important fact has been largely omitted: when KCs undergo their complex lifecycle, they change not only cell size and cell cycle kinetics, but also the actively transcribed RNA content, with the largest RNA content in fresh, actively growing cultured KCs [13]. In microarray, RNA-seq and even qRT-PCR, the same amount of total RNA is loaded for each sample, although yields of the polyA+ RNAs purified from total RNAs may differ. Moreover, normalization for the differential expression test expects equivalent expression levels for several co-expressed genes [14]. Therefore, the genome-wide expression profiling in the previous studies might have underestimated the complexity of the KC transcriptome during their lifecycle.

In this study, we revisit the skin and KC transcriptome with respect to fluctuation of polyA+ RNA content by the keratinocyte statuses; differentiated, activated, senescent and immortalized. Four types of human keratinocyte samples represented these cell statuses: epidermal tissue (split-thickness skin grafts; SGs), cultured primary KCs in early and late passages, and HaCaT cell line. To reduce the sample size and sequencing costs and to control the fluctuation of mRNA concentration, we applied single-cell tagged reverse transcription (STRT) sequencing method for expression profiling using 10 ng of total RNA per sample which is ten times less than required for a conventional RNA-seq method [15]. For accurate expression profiling and statistical tests, we employed STRT RNA-seq with synthetic polyA+ spike-in RNA [16], and SAMstrt statistical package with spike-in based normalization [14]. We first evaluated the improvements of our approach on the genome-wide expression profiling and confirmed the accuracy of the improved methods by literature survey of the keratin and collagen genes. Then we extracted genes that correlated with the sample types, and genes contributing to the sample classification, especially transcription factors [17] and long noncoding RNAs [18], as candidate regulators for keratinocyte characters. These results provide new insights into the skin transcriptome and into the usefulness of primary KCs and HaCaTs as model systems.

## Results and discussion

### Sample preparation, STRT RNAseq, and quality control

**Fig. 1:**
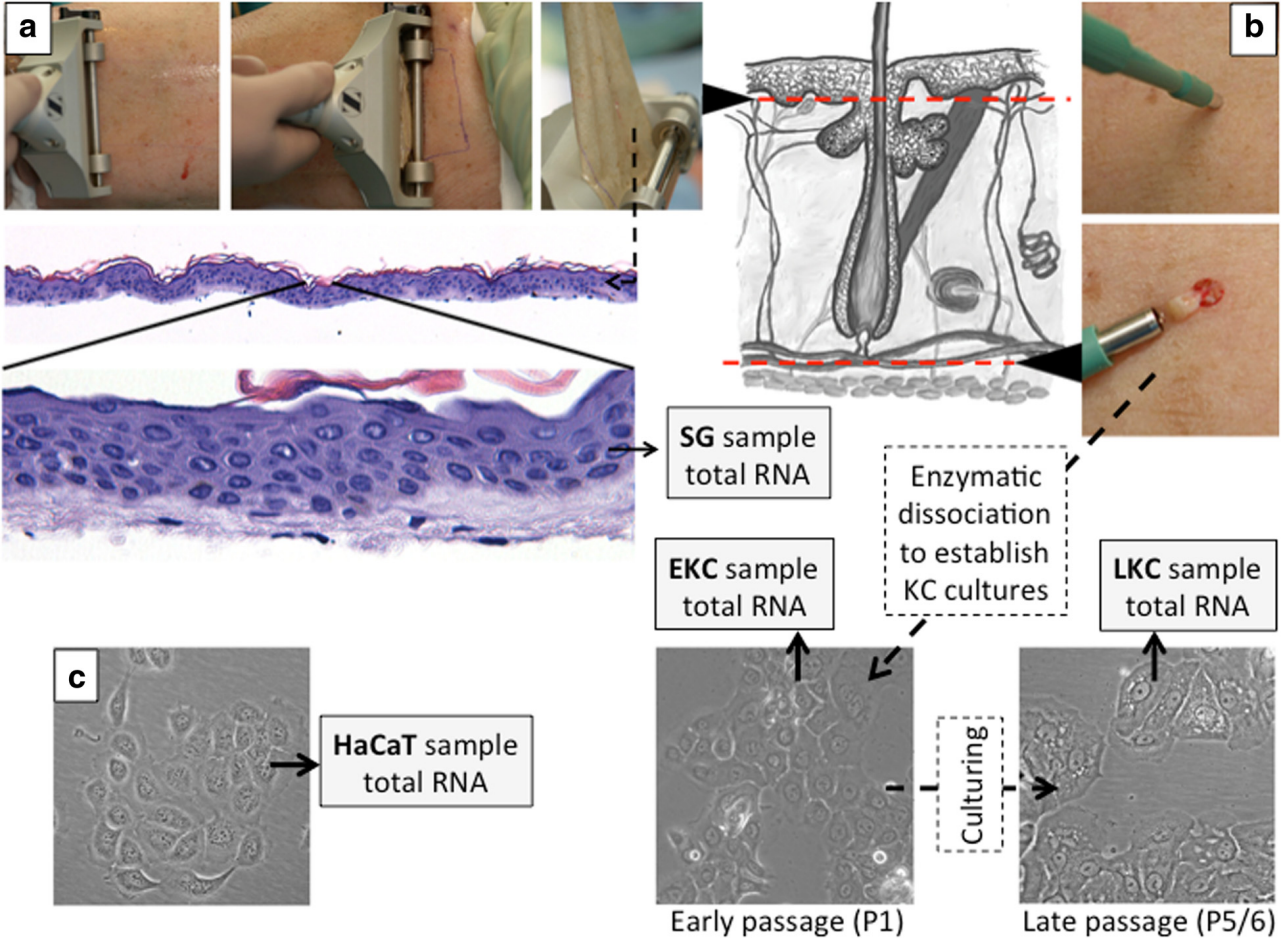
Sample collection. **a** Split-thickness skin graft (SG) samples were harvested in the operating room using a dermatome. Lower panel shows a haematoxylin-eosin stained section of SG sample demonstrating minimal dermis involvement (light blue tissue). **b** Full thickness biopsy samples were collected as 3 mm punch biopsies. Red dotted line in the schematic drawing of skin (middle panel) demonstrates sampling depth for SG samples (upper line) and punch biopsy samples (lower line). Keratinocyte (KC) cultures were established from punch biopsy samples after enzymatic dissociation, and isolated primary KC were cultured to the passage 1 (EKC samples) and to the passage 5/6 (LKC samples). **c** The spontaneously immortalized model keratinocyte cell line, HaCaT, was used as a cell line model. Total RNA was isolated from each sample as outlined in Materials and Methods

The protocol for sample preparation is depicted in Fig. 1. We collected SG biopsies with minimal inclusion of dermis and full thickness skin biopsies for KC culture from 8 donors undergoing plastic surgery (Additional file 1: Table S1). The SG samples were used directly for total RNA extraction whereas the full-thickness biopsies were used to set up KC cultures from which total RNA was extracted at early (1st; EKC) and late (5th-6th; LKC) passages. After RNA quality control (Additional file 2: Table S2), 16 samples were used to prepare two STRT libraries using 3 technical replicates, each containing 10 ng total RNA (Additional file 3: Table S3). Each STRT library was sequenced on four lanes of Illumina HiSeq 2000 instrument. In average, there were 10.8 million STRT reads and 7.92 million mapped reads per replica. After alignment to the human genome and gene-based quantitation, we confirmed consistency between all technical replicates (Additional file 4: Table S4, Additional file 5: Figure S1 and Additional file 6: Figure S2). One sample (10k1b, EKC replica 2 of donor 10k) exhibited exceptionally high relative polyA+ transcript counts (Additional file 5: Figure S1), and displayed unexpected overexpression of some abundantly expressed genes (Additional file 6: Figure S2). Therefore, we excluded it from further consideration as an outlier potentially biasing further analyses.

### Varying polyA+ RNA content in 10 ng total RNA

**Fig. 2:**
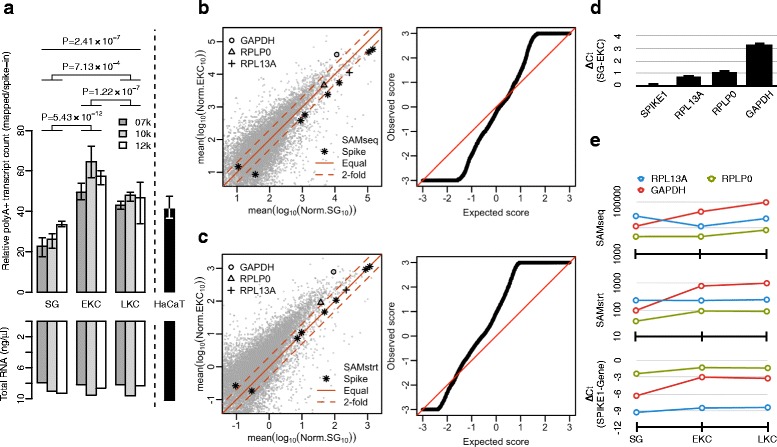
Amounts of polyA+ RNA in cells and effect of normalization. **a** Relative human polyA+ RNA amounts in samples were estimated by relating the human-specific sequence counts to the spike-in sequence counts. The amounts polyA+ RNA detected were EKC>LKC=HaCaT>SG. The EKC samples contained approximately twice as much polyA+ RNA as the SG samples. The samples contained equal concentrations of cellular RNA. **b** Comparison of gene expression between SG and EKCs of donor 10k by applying the gene-based normalization as implemented in SAMseq. Gene expression levels are shown as light gray dots (left panel), levels of spike-in RNAs as asterisks, and three control genes with distinct symbols. The spike-in RNAs appear twofold downregulated, although all samples contained equal amounts of spike-in RNAs. With the gene-based normalization, the Q-Q plot (right panel) shows approximately similar numbers of up- and down-regulated genes, shown as deviations from the diagonal. **c** The same comparison of gene expression between SG and EKCs by applying spike-in normalization as implemented in SAMstrt (left). Spike-in RNA counts follow the diagonal, but many more polyA+ RNAs appear upregulated than downregulated as shown by the upward shift of the gray cloud compared to (b). Q-Q plot (right) shows many more upregulated than downregulated genes, consistent with the increase of relative polyA+ RNA amount as shown in (a). **d** To experimentally validate which normalization yields a more correct analysis, qRT-PCR assays were performed on selected genes. Delta-Ct values indicate no change in spike-in RNA, approximately twofold upregulated RPL13A and RPLP0, and 8-fold upregulated GAPDH, consistent with the spike-in-normalized results. **e** Gene expression profiles for three sample types (SG, EKC, LKC) are similar between SAMstrt-normalized and RT-PCR results, whereas the SAMseq-normalized profiles show mild U shapes for RPL13A and RPLP0, different from the unchanged levels by RT-PCR and SAMstrt

A common assumption is that cells to be compared contain equal amounts of RNA. However, KCs have been shown to change their RNA content over time in long-term culture [13], leading us to further investigate the differences in polyA+ RNA contents in our samples. We found that the estimated polyA+ RNA contents as quantified against the added spike-in RNA controls varied in different sample types (Additional file 5: Figure S1). The variation was larger than the variation of the repeatedly measured total RNA amounts that were loaded for sequencing (Fig. 2a). Such differences can lead to the misinterpretation of differential expression when traditional endogenous gene-based normalization is applied. This is demonstrated in Fig. 2b, which is a comparison of 10k donor samples between SG and EKCs. The endogenous gene-based normalization method did not estimate the spike-in levels equivalently although the amount of spike-in RNAs were equal in all samples. Because the normalized spike-in levels must be same in the comparison, we employed the recently developed normalization method, SAMstrt [14], which uses exclusively the spike-in RNAs for normalization (Fig. 2c). Validation assays by qRT-PCR confirmed the upregulation of two housekeeping genes, *RPLP0* and *RPL13A*, as predicted by the spike-in-based normalization, both of which were predicted unchanged or downregulated by the gene-based normalization (Fig. 2d). Moreover, the spike-in-based normalization method provided more consistent expression patterns in multiple samples with the qRT-PCR measurements (Fig. 2e). In conclusion, the observed variation in the polyA+ RNA content in 10 ng total RNA led to a misinterpretation of the expression pattern by the gene-based normalization method, but it became more reliable by the spike-in based normalization.

### Characterization of SGs, cultured KCs, and HaCaTs by differentially expressed genes

**Fig. 3:**
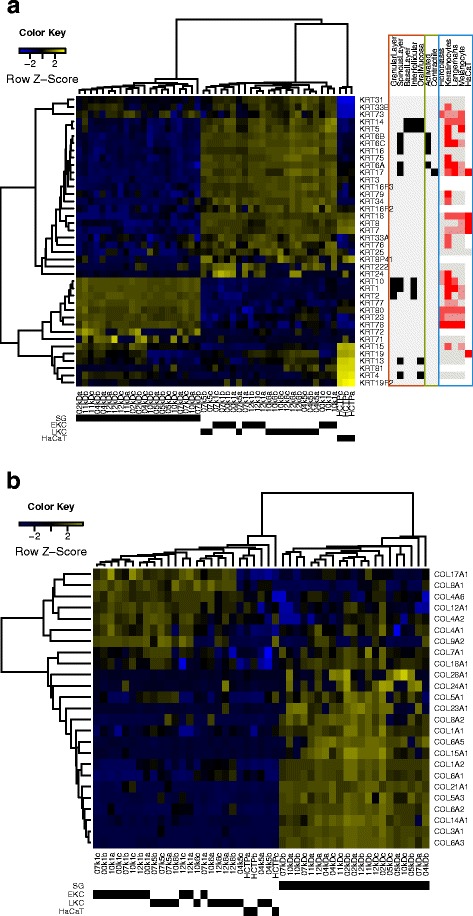
Differential expression between the SGs, cultured KCs and HaCats. **a** The heat map panel to the left shows the expression of cytokeratins in different samples. Annotations for cytokeratins in the panel to the right for the gene expression on different layers and tissue types (orange) are based on Takahashi et al. [19], for the status in activation cycle (green) are based on Freedberg et al. [1], and for the protein expression in five cell types (blue) are based on Human Protein Atlas, HPA [21]. According to HPA, white denotes no data, gray means not detected (primary cell types) or with negative intensity (HaCaT), and red represents varying protein levels according to the intensity of the colour. SG, Split-thickness skin graft; EKC, early passage keratinocyte; LKC, late passage keratinocyte; HCTP, HaCaT. **b** The heat map shows the expression of collagens in different samples

When we assessed the transcriptome profiles in the different samples, we found 11,908 differentially expressed genes (Additional file 7: Table S5). Among them, 40 out of 58 cytokeratin genes were differentially expressed (Fig. 3a), and many of them well known markers for the KC differentiation status both in cell culture and in tissue [1, 19, 20]. Hierarchical clustering confirmed significant contrasts between three sample types: SG, cultured KC and HaCaT. SGs contained several cytokeratin transcripts corresponding to cells at differentiated or differentiating epidermal layers (*KRT1*, *2*, and *10*), whereas the KCs contained cytokeratins typical of cells that maintain their proliferative capacity (*KRT5* and *14*) or that are activated by wound healing, hyperproliferative skin diseases or in vitro culturing (*KRT6*, *16*, and *17*) [1, 20]. *KRT8* and *KRT18* that are the developmentally first keratins absent from normal skin and rather characterizing simple epithelia are in our data expressed by both cultured KCs and HaCaTs supporting their proliferative and undifferentiated nature [20].

There were no significant differences between the early (EKC) and late (LKC) passage KCs in the cytokeratin levels. In contrast, HaCaT was remarkably different from both SGs, EKC and LKC expressing higher levels of cytokeratins 4, 13, 15, 19 and 81 (*KRT4*, *13*, *15*, *19* and *81*). Furthermore, several cytokeratins (*KRT7*, *8*, *18*, and *19*) characterising HaCaT by the spike-in normalization (Fig. 3a) were consistent with published protein expression patterns, low expression in skin, but high in HaCaT [21]. This observation was only partially supported by the gene-based normalization (Additional file 8: Figure S3) – by that method *KRT7* and *8* did not reach significant difference, suggesting that the gene-based normalization was less sensitive or less accurate than the spike-in normalization.

To further confirm the sample classification by STRT results, we compared the sample specific expression of different collagen genes (Fig. 3b), which have previously characterized expression profiles in different types of tissues [22, 23]. 25 out of 44 collagen genes were differentially expressed. SGs expressed mainly connective tissue specific collagens (types I, V and VI), consistent with the presense of thin connective tissue layer underlying the epidermis, whereas cultured cells were characterized by the basement membrane (types IV) and skin hemidesmosome (type XVII) specific collagens again supporting the proliferative nature typical for the KCs in basal layer of epidermis.

### Characterization of SGs, EKCs, LKCs, and HaCaTs by coregulated genes

**Fig. 4:**
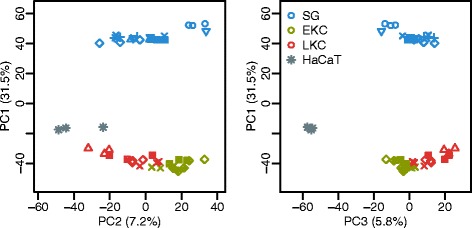
Sample classification by the principal component analysis. PCA scatter plots using the spike-in normalized gene expression profiles. PC1 demonstrates the contrast between SGs and other sample types, PC2 depicts the contrast between EKCs and HaCaTs, and PC3 shows the contrast between HaCaTs and LKCs. Percentages beside of the axis labels are the contribution ratios. Identical symbols between SGs and KCs denote identical donors in three technical replicas each. SG, Split-thickness skin graft; EKC, early passage keratinocyte; LKC, late passage keratinocyte

We approached the question how comparable cultured cells are as model systems for intact skin by elucidating the gene expression differences between the samples. We applied Principal Component Analysis (PCA; Fig. 4) to decompose the differences into several dimensions and to simplify the complexity of our dataset. PCA enabled multiclass comparisons between samples and improved the interpretation of expression profiles. The first principal componene (PC), that depicts the largest variation between samples, classified the SGs as a separate group compared to the other samples. The second and third PCs separated the early and late passages of KCs from HaCaTs, respectively. In the following paragraphs, we interpret the meaning of each PC axis with literature surveys to assess appropriateness of the sample classifications based on our expression profile, and to decode insights into the differentiation and the growth control.

### PC1 demonstrates the contrast between SGs and other sample types

First, we extracted genes correlating with the PC1. In this component, positively correlated genes referred to those with higher expression in the SGs when compared to KCs or HaCaTs, and negatively correlated genes acting oppositely. Our data showed that 3,402 genes correlated positively with the PC1 and thus were expressed higher in SGs (Table 1a and Additional file 9: Table S6), although the total polyA+ content of SGs was lower than that of KCs and HaCaTs. In contrast, 4,663 genes correlated negatively (Table 1a and Additional file 9: Table S6). Nine out of ten most upregulated genes in the SGs were previously associated with epidermal differentiation or with small organelles in differentiating cells (Table 1a), and five of ten most upregulated genes in KCs and HaCaTs were annotated as localized in mitochondria (Table 1b), which are lost from keratinocytes during epidermal differentiation [24]. To conclude, the most correlated known genes contributing to PC1 were thus consistent with the biological phenotype of the contrasted cell types.

**Table 1:** Ten most correlated genes with PC1. Tables a and b are subsets of significantly correlated genes with relevant functions in skin lineage (for full data, see Additional file 9: Table S6). a Positively correlated genes, which were upregulated in SGs, and (b) negatively correlated genes, which were upregulated in KCs and HaCaTs

Gene Symbol	Score	Local FDR	
(a)			
RAB11FIP1	0.934	0.00044	
KRT80	0.933	0.00045	Localization around desmosomal plaques in earlier stages of differentiation [PMID:20843789]
ID4	0.915	0.00052	Lack of the protein in parakeratotic cells at upper skin layer [PMID:21663940]
PPL	0.911	0.00053	A component of desmosomes and of the cornified envelope [PMID:9412476]
KRT1	0.911	0.00054	Specifically expressed in the spinous and granular layers [PMID:10511477]
BCL6	0.900	0.00058	Expression at the terminal differentiation stage [PMID:8912662]
ERBB3	0.897	0.00059	Skin biopsy expressed more than the cultured cells [PMID:11571634]
PLAC2	0.896	0.00059	lncRNA controlling terminal differentiation [PMID:23201690]
PKP1	0.896	0.00059	Localization around desmosomal plaques and nuclei [PMID:16632867]
KRT10	0.894	0.00060	Specifically expressed in the spinous and granular layers [PMID:10511477]
(b)			
PRDX3	−0.922	0.00044	Mitochondrial [PMID:17893648]
SEC61G	−0.914	0.00045	
NDUFB3	−0.914	0.00052	Mitochondrial [PMID:12611891]
FXC1	−0.913	0.00053	Mitochondrial [PMID:14726512]
ATP5J	−0.911	0.00054	Mitochondrial [PMID:12110673]
GNG10	−0.911	0.00058	
TXNDC9	−0.911	0.00059	
MRPS23	−0.910	0.00059	Mitochondrial ribosomal protein
ENY2	−0.910	0.00059	
PSMB2	−0.909	0.00060	

To further interpret the PC1 and to find the associations between genes and phenotypes contrasted on PC, we performed gene set enrichment analysis amongst all genes that correlated with that PC (PC-GSEA). Our results showed that 136 out of 7,801 gene sets correlated positively, being upregulated in the SGs (q-value FDR < 1 %; Additional file 10: Table S7). The most significant gene set (Table 2a) was target genes of p53 and p63, which are known mediators of keratinocyte differentiation [25]. Similarly, 1,340 gene sets showed negative correlation (q-value FDR < 1 %; Additional file 10: Table S7). The 2nd, 4th, 6th, 9th, and 10th of significant gene sets (Table 2b) were related to mitochondria. Therefore, the PC-GSEA extracted meaningful contrasts of the biological functions and the phenotypes to interpret the PC1.

**Table 2:** Ten most significant gene sets enriched in PC1 correlating genes. Tables a and b are subsets of gene sets that were significantly (FDR q-value < 1 %) enriched in PC1 correlating genes by PC-GSEA (for full data, see Additional file 10: Table S7). (a) Gene sets in positively correlated genes, which were upregulated in SGs, and (b) gene sets in negatively correlated genes, which were upregulated in KCs and HaCaTs. SIZE is number of genes belonging the gene set. NES is normalized enrichment score

Name	SIZE	NES	FDR q-val
(a)			
PEREZ_TP53_AND_TP63_TARGETS	198	2.967	0
SMID_BREAST_CANCER_NORMAL_LIKE_UP	449	2.908	0
BOQUEST_STEM_CELL_CULTURED_VS_FRESH_DN	31	2.774	0
KRAS.LUNG_UP.V1_DN	128	2.774	0
ABATES_COLORECTAL_ADENOMA_DN	248	2.760	0
WU_SILENCED_BY_METHYLATION_IN_BLADDER_CANCER	53	2.758	0
PEREZ_TP63_TARGETS	334	2.742	0
WINNEPENNINCKX_MELANOMA_METASTASIS_DN	46	2.735	0
KEGG_ASTHMA	23	2.689	0
KEGG_ALLOGRAFT_REJECTION	33	2.663	0
(b)			
YAO_TEMPORAL_RESPONSE_TO_PROGESTERONE_CLUSTER_13	172	3.060	0
MOOTHA_VOXPHOS	86	3.038	0
WONG_EMBRYONIC_STEM_CELL_CORE	332	3.028	0
REACTOME_RESPIRATORY_ELECTRON_TRANSPORT_ATP_SYNTH ESIS_BY_CHEMIOSMOTIC_COUPLING_AND_HEAT_PRODUCTION_ BY_UNCOUPLING_PROTEINS_	81	3.016	0
FOURNIER_ACINAR_DEVELOPMENT_LATE_2	276	3.005	0
KEGG_OXIDATIVE_PHOSPHORYLATION	111	2.954	0
PENG_LEUCINE_DEPRIVATION_DN	186	2.947	0
GSE22886_UNSTIM_VS_IL15_STIM_NKCELL_DN	199	2.947	0
WONG_MITOCHONDRIA_GENE_MODULE	216	2.943	0
REACTOME_RESPIRATORY_ELECTRON_TRANSPORT	65	2.924	0

**Fig. 5:**
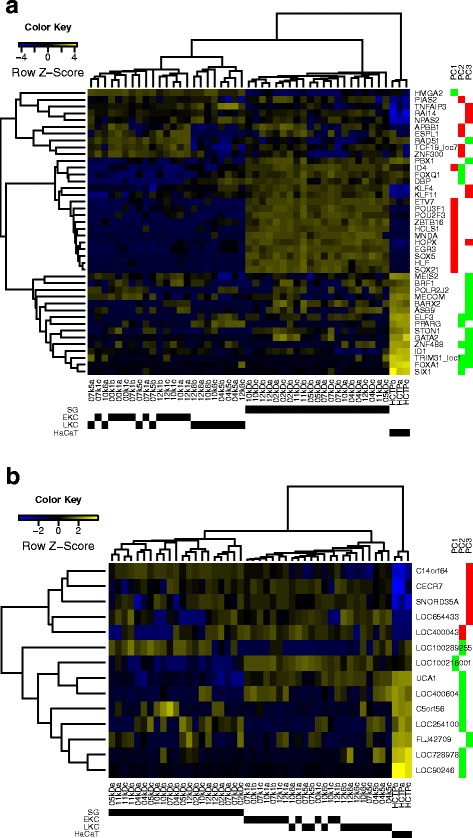
Hierarchical clustering of expression profiles of high loading loci contributing to the sample classification for PC1, PC2 and PC3. Panel (**a**) shows transcription factors (TFs) in the high loading features, and panel (**b**) known noncoding genes. The expression of different TFs separates SGs, KCs and HaCaTs from each other whereas the expression of noncoding genes mainly separates HaCaTs from other samples. The contribution to the three first PCs is shown to the right red denoting positively contributing genes and green denoting negatively contributing genes. SG, Split-thickness skin graft; EKC, early passage keratinocyte; LKC, late passage keratinocyte; HCTP, HaCaT

Since the PC scores representing the functions and phenotypes were calculated by linear combination of the expression profile and the loading coefficients, genes with large loading coefficients on each PC would be the key regulators for the functional contrast. For example, in case of PC1, genes with high positive loading coefficients contribute to the characteristic phenotypes and functions of SGs, and conversely, genes with high negative loading coefficients contribute to common characteristic phenotypes and functions of HaCaTs and KCs. When we extracted genes contributing to PC1, 223 genes showed positive loading and 104 genes showed negative loading (three sigma; Additional file 11: Table S8). 12 of the 223 positively contributing genes that explain the functions in SGs, and one of the 104 negative ones, characterizing the cultured cells, were known transcription factors (Fig. 5a; the definition of a transcription factor was based on [26]). Six of the 12 transcription factors upregulated in SGs were known regulators for skin maturation and differentiation phenotypes (Table 3). As an example, *POU2F3* (a.k.a. *Skn-1a*/*Oct11*) is a known transactivator of the suprabasal layer marker *KRT10* [27], which correlated positively with PC1 (Score = 0.89, local-FDR = 6.00 × 10^−4^), and is also a known repressor for the basal layer marker *KRT14* [28], which correlated negatively with PC1 (Score = −0.68, local-FDR ~ 0). To conclude, differentiation and the mitochondrial phenotypes are possible interpretations of the PC1 and can be explained by the fluctuation of transcription factors that were clearly associated to sample types.

**Table 3:** Transcription factors, positively contributing to PC1. Table shows a subset of significantly contributing loci with relevant functions in skin lineage (for full data, see Additional file 11: Table S8)

Gene Symbol	Loading	Z-score	
HLF	1.059	3.690	Inhibitor of cell death response [PMID:23415677]
ZBTB16	1.039	3.621	
HOPX	1.022	3.558	Controlling differentiation-dependent genes [PMID:20226564]
EGR3	0.999	3.477	
ID4	0.986	3.436	Lack of the protein in parakeratotic cells at upper skin layer [PMID:21663940]
MNDA	0.968	3.369	
HCLS1	0.947	3.297	
SOX5	0.947	3.295	Genes coding SOX5 binding sites at the promoters were PC1 positive correlation (V$SOX5_01; q-value FDR ~ 0)
SOX21	0.937	3.261	Master regulator of hair shaft cuticle differentiation [PMID:19470461]
ETV7	0.928	3.227	
POU3F1	0.920	3.200	Transactivator for FLG (a.k.a. profilaggrin; PC1 positive correlation, Local- FDR = 7.34 × 10-4; [PMID:10809764])
POU2F3	0.906	3.151	10-4; [PMID:10809764] & [PMID:7682011]); repressor for KRT14 (PC1 negative correlation, Local-FDR ~ 0; [PMID:11429405])

### PC2 depicts the contrast between EKCs and HaCaTs

PC2 captured the second largest variation which mostly shows the contrast between HaCaTs and EKCs, with intermediate LKCs (Fig. 4). Three thousand nine hundred sixty nine genes correlated positively with PC2 and were thus expressed at higher level in EKCs (Additional file 9: Table S6), and six of the top 10 positively correlated genes were polyA+ RNA binding proteins (Table 4). In contrast, there were no negatively correlated genes that would appear upregulated in HaCaT, which may depend on the fact that HaCaTs and LKCs had less polyA+ RNA than EKCs (Fig. 2a). Accordingly, PC-GSEA towards PC2 revealed 23 positively correlating and only two negatively correlating gene sets (Table 5 and Additional file 10: Table S7). When we investigated associations in the correlating genes and the gene sets as an interpretation of PC2, we found that the PC2 explained the difference of G1/S-transition between the EKCs and HaCaTs both through negative and positive correlation. First, the genes that bear H3K27me3 in ES cells and have high-CpG-density promoter, showed negative correlation (i.e. upregulation in HaCaTs; MIKKELSEN_ES_HCP_WITH_H3K27ME3; q-value FDR ~ 0) [29]; the genes with H3K27me3 marks have actually been shown to be transcribed at G1/S- and S-phases in HaCaTs. Second, target genes of *RB1*, which is known to be negative regulator of the S-phase entry, showed positive correlation (i.e. upregulation in EKCs; EGUCHI_CELL_CYCLE_RB1_TARGETS, q-value FDR = 4.76 × 10^−3^) [30].

**Table 4:** Ten most positively correlated genes with PC2. Table is a subset of positively correlated genes with PC2, and the thick mark indicates if the gene codes polyA+ RNA binding protein (for full data, see Additional file 10: Table S6)

Gene symbol	Score	Local FDR	polyA + RNA binding [PMID: 22658674]
HNRNPH1	0.855	0.00000	✓
SCAF11	0.829	0.00002	✓
HNRNPA3	0.823	0.00003	✓
BCLAF1	0.802	0.00008	✓
OXCT1	0.800	0.00008	✓
ODF2L	0.798	0.00008	✓
HNRNPAB	0.789	0.00010	✓
PNN	0.788	0.00011	✓
UBE2G2	0.787	0.00011	✓
SPARC	0.787	0.00011	✓

**Table 5:** Most significant gene sets enriched in PC2 correlating genes. (a) Table is a subset of gene sets (ten out of 23) significantly (FDR q-value < 1 %) enriched in PC2 positively correlating genes, which were upregulated in EKCs, by PC-GSEA (for full data, see Additional file 10: Table S7). (b) Table is gene sets significantly enriched in PC2 negatively correlating genes, which were upregulated in HaCaTs. SIZE is number of genes belonging the gene set. NES is normalized enrichment score

Name	SIZE	NES	FDR q-val
(a)			
PUJANA_BRCA_CENTERED_NETWORK	117	1.909	0.00492
ZHANG_TLX_TARGETS_UP	89	1.875	0.00885
BURTON_ADIPOGENESIS_PEAK_AT_16H	40	1.873	0.00590
PUJANA_XPRSS_INT_NETWORK	168	1.869	0.00467
ZHANG_TLX_TARGETS_36HR_DN	185	1.868	0.00394
EGUCHI_CELL_CYCLE_RB1_TARGETS	23	1.856	0.00476
REACTOME_TRANSPORT_OF_MATURE_TRANSCRIPT_TO_CYTOPLASM	52	1.846	0.00436
ABRAMSON_INTERACT_WITH_AIRE	44	1.845	0.00394
GOLUB_ALL_VS_AML_UP	24	1.841	0.00383
ZHANG_TLX_TARGETS_60HR_DN	275	1.839	0.00374
(b)			
MIKKELSEN_ES_HCP_WITH_H3K27ME3	35	2.441	0.00000
REACTOME_XENOBIOTICS	15	2.345	0.00851

Then we proceeded to find genes that are the key regulators for the functional contrast on PC2. 137 genes showed high positive loading to PC2, and 111 genes showed negative loading (three sigma; Additional file 11: Table S8). Among them, five positive loading genes and nine negative ones were transcription factors (Fig. 5a). Interestingly, the positive loading genes *APBB1* [31], *ESPL1* [32], *TCF19* [33] and *ZNF300* [34] were known cell cycle regulators or contributors to cell proliferation. Moreover, one of the 137 positive loading genes and seven of the 111 negative ones were known lncRNAs (Fig. 5b). *UCA1* was a negatively loading lncRNA, known as essential for bladder cancer cell proliferation via CREB-dependent pathway [35]. Interestingly, Cyclin D1 expression in KCs is also under the CREB-dependent pathway [36]. In conclusion, the difference of G1/S-transition between HaCaTs and EKCs is a possible interpretation for PC2, and again the fluctuation of transcription factors was highly associated to sample types.

### PC3 shows the contrast between HaCaTs and LKCs

PC3 associated mostly with the variation between HaCaTs and LKCs (Fig. 4). We could not detect positively correlated genes (upregulated in LKC) with PC3, although these samples had equivalent polyA+ RNA contents (Fig. 2a). In contrast, 2,992 correlated negatively refering to upregulation in HaCaT (Additional file 9: Table S6), and five of the top 10 negatively correlated genes codes polyA+ RNA binding protein (Table 6). In PC-GSEA, only 30 gene sets correlated positively with PC3, whereas 241 correlated negatively (Additional file 10: Table S7). As the interpretation of PC3, we found that it was associated with senescense, in accordance of KC senescence that does not apply to the continuously proliferating HaCaT cells. One explaining component was the positive correlation (upregulation in LKCs) of potassium channel genes (Table 7a). Potassium channel activation inhibits proliferation by activating a senescence program in breast cancer [37], and the G0/G1-arrest is accompanied by this activation. Consistently, not only the genes for G0 and early G1 phases, but also the other cell cycle associated genes were downregulated in LKCs (Table 7b). Furthermore, *miR-192* and *miR-34* target genes were upregulated in HaCaTs (negative correlation with PC3; Table 7c). Those miRNAs are functionally associated with p53-dependent cellular maintenance and aging (miR-34 [38, 39], and miR-192 [40, 41]).

**Table 6:** Ten most negatively correlated genes with PC3. Table is a subset of negatively correlated genes with PC3, and the thick mark indicates if the gene codes polyA+ RNA binding protein (for full data, see Additional file 9: Table S6)

Gene symbol	Score	Local FDR	polyA + RNA binding [PMID: 22658674]
PRPF8	−0.885	0.00006	✓
APOL6	−0.826	0.00018	
CCDC15	−0.826	0.00018	
TTF1	−0.823	0.00019	
ZC3H11A	−0.813	0.00021	✓
NUCKS1	−0.809	0.00021	✓
PCBP2	−0.808	0.00021	✓
H2AFV	−0.804	0.00022	
RBBP7	−0.500	0.00023	
HMGN2	−0.798	0.00023	✓

**Table 7:** Significant gene sets enriched in PC3 correlating genes. Tables a, b and c are subsets of gene sets significantly (FDR q-value < 1 %) enriched in PC3 correlating genes by PC-GSEA (for full data, see Additional file 10: Table S7). (a) Table is a subset of the ten (out of 30) gene sets most significantly enriched in the positively correlating genes, which were upregulated in LKCs. (b) Table is cell cycle associated gene sets significantly enriched in the negatively correlating genes, which were upregulated in HaCaTs. (c) Table is miRNA-target gene sets significantly enriched in PC3 negatively correlating genes. SIZE is number of genes belonging the gene set. NES is normalized enrichment score

Name	SIZE	NES	FDR q-val
(a)			
REACTOME_OLFACTORY_SIGNALING_PATHWAY	36	2.967	0
DAZARD_UV_RESPONSE_CLUSTER_G28	20	2.908	0
DAZARD_UV_RESPONSE_CLUSTER_G24	27	2.774	0
PID_CONE_PATHWAY	16	2.774	0
MAHADEVAN_RESPONSE_TO_MP470_DN	19	2.760	0
WANG_TNF_TARGETS	23	2.758	0
BURTON_ADIPOGENESIS_1	33	2.742	0
AMIT_EGF_RESPONSE_60_MCF10A	38	2.735	0
VOLTAGE_GATED_POTASSIUM_CHANNEL_COMPLEX	29	2.689	0
RORIE_TARGETS_OF_EWSR1_FLI1_FUSION_UP	30	2.663	0
(b)			
REACTOME_G2_M_CHECKPOINTS	41	1.865	0.00029
REACTOME_MITOTIC_PROMETAPHASE	85	1.822	0.00064
REACTOME_MITOTIC_M_M_G1_PHASES	167	1.783	0.00114
REACTOME_G0_AND_EARLY_G1	23	1.732	0.00241
REACTOME_CELL_CYCLE	392	1.710	0.00314
REACTOME_CELL_CYCLE_CHECKPOINTS	111	1.690	0.00428
REACTOME_S_PHASE	106	1.660	0.00596
REACTOME_M_G1_TRANSITION	78	1.646	0.00706
REACTOME_REGULATION_OF_MITOTIC_CELL_CYCLE	77	1.621	0.00908
(c)			
GEORGES_CELL_CYCLE_MIR192_TARGETS	62	1.726	0.00272
TOYOTA_TARGETS_OF_MIR34B_AND_MIR34C	449	1.675	0.00501

We next attempted to find supporting genes for this interpretation. A total of 117 genes showed high positive loading to PC3, and 144 genes showed high negative loading (three sigma; Additional file 11: Table S8). Among them, six positive and 15 negative loading genes were transcription factors (Fig. 5a). One negatively loading (upregulated in HaCaTs) gene was *RAD51*, known to be involved in the homologous recombination and the repair of DNA. Expression of *RAD51* is regulated by Lamin A (*LMNA*) [42], and expression of the *LMNA* was consistently negatively correlated (Local-FDR = 8.99 × 10^−4^). It’s well known that the mutations of *LMNA* lead to Hutchinson–Gilford progeria syndrome characterized by a premature aging [43]. Therefore, senescence and cellular aging responces would be a possible interpretation for PC3.

## Conclusion

STRT RNA-seq method complemented with synthetic RNAs revealed a variation of polyA+ RNA content per total RNA in different cell types, SGs, EKCs, LKCs and HaCaTs, reflecting the activity of the cell type. Even though the STRT reads are concentrated towards the 5’end of the polyA+ transcripts and the method has limited resolution at 2nd and more downstream exons, the advantages of the method include the small amount of starting material needed for library preparation, early multiplexing of up to 92 samples reducing the cost and time of library preparation, and the inclusion of external spike-in RNAs as a standard procedure. The spike-in normalization has been shown to be a valuable tool when comparing samples with fluctuating polyA+ RNA contents [14, 44]. We showed that the use of spike-in-based normalization produced consistent results with qPCR validations, and provided us with deeper insights into KC biology. In contrary, the traditional gene-based normalization method led to inaccurate expression profiles. Moreover, our approach would be applicable not only for the studies on KCs but also for the other studies with fluctuation of polyA+ RNA content, for example those on single cells with different types or sizes [45, 46].

We applied PCA to elucidate dissimilarity between the samples, and also to decompose the differences. The three first PCs represented differentiation and the mitochondrial phenotypes between SGs and cultured cells, G1/S-transition between HaCaTs and EKCs, and senescence and cellular aging responces between HaCaTs and LKCs. All cultured cells differed from tissue samples and HaCaT cells differed remarkably from other cultured cells based on both PCA and the comparison of previously known KC markers, cytokeratins. Our results thus suggest that great caution should be payed when using cultured primary KCs and cell models like HaCaTs as models for skin, especially when focusing on the pathways revealed by PCA. The transcriptomes of cultured primary KCs and HaCaTs resemble that of acivated skin rather than normal skin as shown also by others [10, 11].

In this study, we present an approach to compare highly varying cell types by applying synthetic RNA based normalization. For further studies or applications of other biological events, the key is to find the hidden associations between genes and phenotypes which are contrasted on PCs. We also note that there are still many poorly annotated genes in the genome that might be revealed by our approach. Moreover, PCA without sample pre-classification might be applied in studying gene expression in complex disorders using a large enough cohort.

## Methods

### Sample collection, cell culture, RNA extraction, and STRT RNA-seq

All subjects involved in this study provided written informed consent under a protocol adherent to the Helsinki Guidelines and approved by the Institutional Review Board of the Helsinki University Central Hospital.

Split-thickness skin grafts (SGs) were harvested as outlined previously [47]. Briefly, SG samples were obtained using a compressed air-driven dermatome (Zimmer®, Warsaw, IN) with a fixed thickness setting of 2/1000 in. to obtain a representative sample of epidermis to its full thickness with minimal dermis involvement from the donor site skin. In order to initiate keratinocyte cultures, full thickness skin samples (3-mm diameter punch biopsies) were collected (Additional file 1: Table S1). The quality of SG samples was examined from HE-stained paraffin sections. The SGs were immediately immersed in RNAlater to ensure the least possible manipulation and gene expression changes.

Epidermal cells were isolated from the full thickness skin with dispase digestion followed by trypsinization to enable collection of all primary cell types and phenotypes of the epidermis in the initial harvest. Human primary keratinocytes were cultured in Keratinocyte Growth Medium 2 (PromoCell # C-20011) with calcium (0.06 mM) and PromoCell supplements (#C-39016). Cell culture dishes were coated with collagen I (Gibco Rat tail, A-10483-01). Cells were routinely passaged, and samples were collected from early (passage 1; EKC) and late passages (passage 5 or 6; LKC). The human immortalized keratinocyte HaCaT cell line was grown in low glucose DMEM (Lonza) supplemented with 5 % FBS, 2 mM L-glutamine, 1 mM sodium pyruvate solution, 0.1 mM non-essential amino acids, 100 U/ml penicillin and 100 μg/ml streptomycin at 37 °C and 5 % CO2. HaCaT cells were collected at passage 43 for RNA isolation.

Total RNA was extracted by miRNeasy kit (Qiagen) from both tissue samples and cells. RNA concentrations were measured by Nanodrop and Qubit and the quality was controlled by Bioanalyzer (RIN for all samples >8; Additional file 2: Table S2).

### STRT RNA-seq

Qualified total RNA samples (10 ng of each replicate, three replicates for each sample) were used for RNA sequencing library preparation according to the STRT protocol [16], which was adjusted for 10 ng samples by decreasing the number of cycles to 10 during the first PCR amplification. The libraries (Additional file 3: Table S3) were sequenced using an Illumina HiSeq 2000 instrument. Preprocessing of STRT reads, alignments and per-gene quantitation were performed by an established analysis pipeline [16]. Differential expression analysis was performed by SAMstrt [14], and also by SAMseq [48] for an example of the gene-based normalization. Differentially expressed genes by the four sample types were extracted by multiclass response test; threshold of the significantly regulated genes is Local-FDR < 1 %. Gene type (e.g. protein coding, pseudo, noncoding), genes of glycolysis/gluconeogenesis and transcription factors were classified based on Entrez Gene, KEGG pathway [KEGG:hsa00010] and Ravasi et al. [26], respectively. For cytokeratin expression analysis, we selected the gene symbols that begin “KRT” followed by number. We performed PCA with the scaling but non-centering preprocess steps. Correlation of gene expression with PC was estimated by SAMstrt quantitative response test. Scores of samples on a PC were given as the quantitative values, and threshold of the significantly correlated gene is Local-FDR < 1 %. PC-GSEA, which tests correlation of gene set with PC, was by GSEA [49] preranked test towards c1, c2, c3, c5, c6 and c7 of MSigDB version 4. The ranked lists contain the gene correlation scores estimated by SAMstrt as descrived above; threshold of the significantly correlated gene set is q-value FDR < 1 %.

### qRT-PCR

The qualified total RNA samples 10kD, 10k1 and 10k6, which were used for sequencing with STRT method, were subjected to cDNA synthesis. Equal amount of the SPIKE-in RNA mix (ArrayControl RNA, AM1780M, Ambion) was added to each cDNA synthesis reaction according to the STRT-library preparation protocol described above. cDNA synthesis was carried out using oligo dT-primers and SuperScript III First-Strand synthesis system for RT-PCR (18080–151, Invitrogen) according to manufacturer’s instructions. 5 ng of cDNA was applied to each qPCR assay and each sample was run in three technical replicates. qPCR was carried out using an ABI PRISM 7500 Fast Real-Time PCR System with Fast SYBR® Green Master mix (4385617, both from Applied Biosystems) according to manufacturer’s instructions. The primer sequences are shown in Additional file 12: Table S9.

## Availability of supporting data

The processed STRT reads supporting the results of this article are available in the European Nucleotide Archive (http://www.ebi.ac.uk/ena/data/view/PRJEB8997).

## Additional files

Additional file 1: Table S1. Patients and the prepared samples. Additional file 2: Table S2. The prepared samples and the total RNA qualities. Additional file 3: Table S3. STRT library designs, sample notation and the sequencing summary. The sample name consists of 5 characters. The former 4 characters represent the sample type whether HaCaT cell line (HCTP) or the other primary samples as defined by patient and type identifiers in Additional file 2: Table S2. The last one character either a, b or c represent the technical replicas. Additional file 4: Table S4. Similarity between technical replicates. The similarity was estimated by Pearson correlation coefficients of raw read counts. Additional file 5: Figure S1. Variation of relative polyA+ transcript counts. There are three replicas (a, b and c from dark to bright filling colors) in each sample. The relative polyA+ transcript count was estimated by mapped read count per spike-in read count; we added the same amount of polyA+ tailed spike-in RNAs into all 10 ng total RNA samples before the library synthesis. The ratio of the mapped reads on the reference human genome to the spike-in reads is a relative concentration of endogenous polyA+ RNAs in the 10 ng total RNA, which can be compared with the other samples. One outlier, 10k1b, was excluded for the further analysis as described in Additional file 6: Figure S2. Additional file 6: Figure S2. Unexpected overexpression of some abundantly expressed genes in 10k1b. Scatter plots show comparisons of per-gene raw read counts between the technical replicas. (a) Comparisons in 07kD SGs as a reference. These replicas had the worst correlation coefficients (see also Additional file 4: Table S4). (b) Comparisons in 10k1 EKCs. The comparisons with the replica b, especially between the replicas a and b, had broader scatter than one of replicas a vs. c and the 07kD replicas. The 10k1b is the only outlier in the replicas. Heat maps and the hierarchical clustering of significantly expressed cytokeratins (c) and glycolysis-gluconeogenesis (d) genes including the 10k1b replica. Some genes of the 10k1b were overrepresented, although the trends were similar. Additional file 7: Table S5. Differentially expressed genes between the multiple classes. There are 11,908 unique genes in 12,246 (of 20,500) differentially expressed features. The columns from G to L are the statistical values estimated by SAMstrt. Local Gene ID is usually identical with Gene Symbol except for multicopy genes, which have suffix “_loc” with numbers, and for repetitive elements, which have prefix “r_”. Entrez Gene ID and Gene Type are based on Entrez Gene. Glycolysis genes and Transcription Factors are denoted “GL” and “TF” at the columns E and F. Contrasts are the standardized mean difference of the gene expression between the four classes. Positive contrast means overexpression, and negative contrast the opposite. Additional file 8: Figure S3. Hierarchical clustering of cytokeratin expression profiles with gene-based normalization. In the 11,689 differentially expressed genes by the gene-based normalization, 38 were known cytokeratin genes shown in this figure. The heat map panel to the left shows the expression of cytokeratins in different samples. Annotations for cytokeratins in the panel to the right for the gene expression on different layers and tissue types (orange) are based on Takahashi et al. [19], for the status in activation cycle (green) are based on Freedberg et al. [1], and for the protein expression in five cell types (blue) are based on Human Protein Atlas, HPA [21]. According to HPA, white denotes no data, gray means not detected (primary cell types) or with negative intensity (HaCaT), and red represents varying protein levels according to the intensity of the colour. Additional file 9: Table S6. PC correlating loci. Sheet name indicates PC number and direction of the correlation. Each sheet contains significantly correlated loci (Local-FDR < 1 % by SAMstrt quantitative response test towards the score of samples on the PC; the columns C and D are the statistical values). Local Gene ID is usually identical with Gene Symbol except for multicopy genes, which have suffix “_loc” and the copy number. There were no negatively correlating genes with PC2 and no positively correlating genes with PC3. Additional file 10: Table S7. PC correlating gene sets. Sheet name indicates PC number and direction of the correlation. Each sheet contains significantly correlating gene sets (q-value FDR < 1 % by GSEA [49] with a gene list preranked by SAMstrt quantitative response test towards the score of samples on PC). Additional file 11: Table S8. PC contributing features. Sheet name indicates PC number and direction of the contribution. Each sheet contains significantly high contributing loci (three sigma). Local Gene ID is usually identical with Gene Symbol except for multicopy genes, which have suffix “_loc” and the copy number. Additional file 12: Table S9. qRT-PCR primers.

## Abbreviations

EKC: Cultured keratinocyte at early passage
GSEA: Gene set enrichment analysis
KC: Keratinocyte
LKC: Cultured keratinocyte at late passage
PC: Principal component
PCA: Principal component analysis
SG: Split-thickness skin graft
STRT: Single-cell tagged reverse transcription
