# Supplementary figures and images for: Gene expression analysis of skin grafts and cultured keratinocytes using synthetic RNA normalization reveals insights into differentiation and growth control
Source: BMC Genomics. 2015 Jun 25;16(1):476. doi: 10.1186/s12864-015-1671-5 (PMC4480911; doi:10.1186/s12864-015-1671-5)

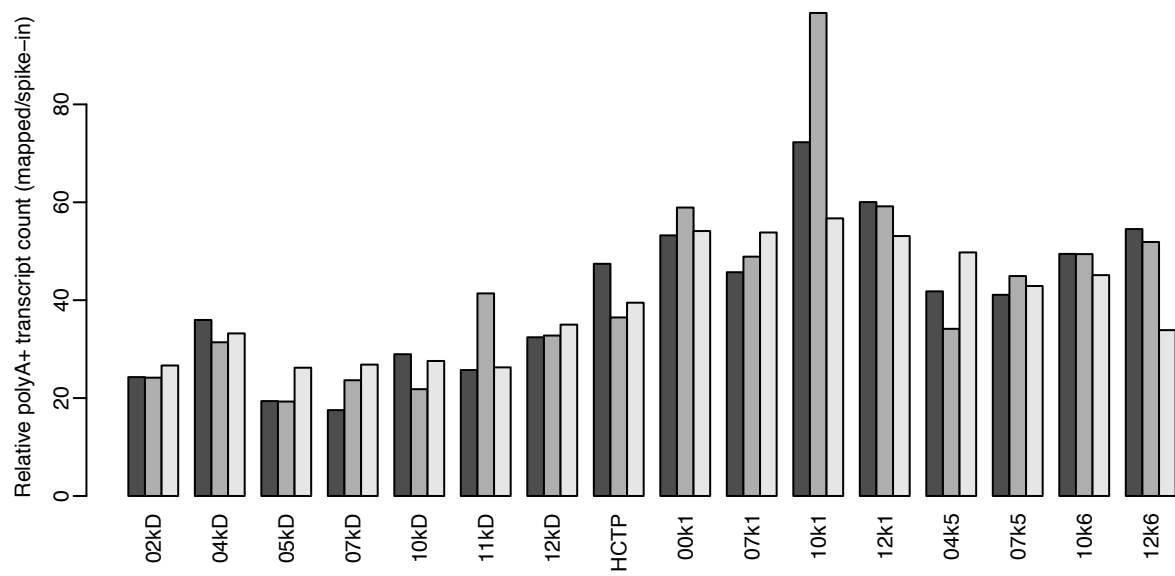

Figure S1

Supplement: Additional file 5: Figure S1. — Variation of relative polyA+ transcript counts. There are three replicas (a, b and c from dark to bright filling colors) in each sample. The relative polyA+ transcript count was estimated by mapped read count per spike-in read count; we added the same amount of polyA+ tailed spike-in RNAs into all 10 ng total RNA samples before the library synthesis. The ratio of the mapped reads on the reference human genome to the spike-in reads is a relative concentration of endogenous polyA+ RNAs in the 10 ng total RNA, which can be compared with the other samples. One outlier, 10k1b, was excluded for the further analysis as described in Additional file 6: Figure S2. [file 12864_2015_1671_MOESM5_ESM.pdf]

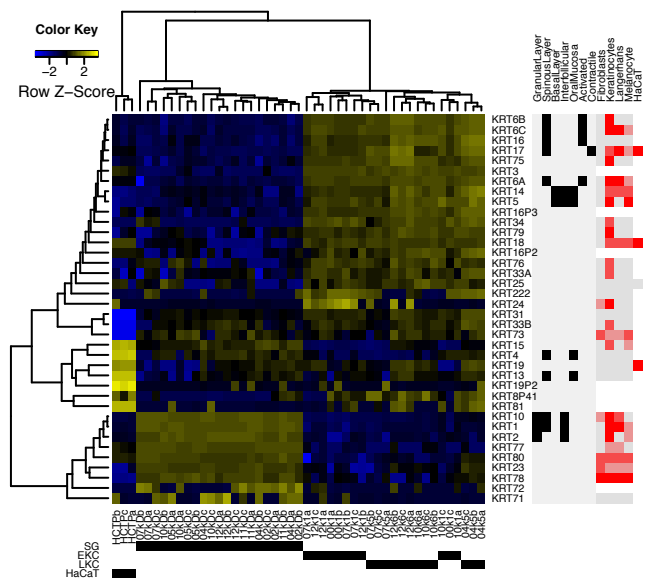

Figure S3

Supplement: Additional file 8: Figure S3. — Hierarchical clustering of cytokeratin expression profiles with gene-based normalization. In the 11,689 differentially expressed genes by the gene-based normalization, 38 were known cytokeratin genes shown in this figure. The heat map panel to the left shows the expression of cytokeratins in different samples. Annotations for cytokeratins in the panel to the right for the gene expression on different layers and tissue types (orange) are based on Takahashi et al. [19], for the status in activation cycle (green) are based on Freedberg et al. [1], and for the protein expression in five cell types (blue) are based on Human Protein Atlas, HPA [21]. According to HPA, white denotes no data, gray means not detected (primary cell types) or with negative intensity (HaCaT), and red represents varying protein levels according to the intensity of the colour. [file 12864_2015_1671_MOESM8_ESM.pdf]
