# Supplementary material for: Gene expression analysis of skin grafts and cultured keratinocytes using synthetic RNA normalization reveals insights into differentiation and growth control
Source: BMC Genomics. 2015 Jun 25;16(1):476. doi: 10.1186/s12864-015-1671-5 (PMC4480911; doi:10.1186/s12864-015-1671-5)
Supplement: Additional file 6: Figure S2. — Unexpected overexpression of some abundantly expressed genes in 10k1b. Scatter plots show comparisons of per-gene raw read counts between the technical replicas. (a) Comparisons in 07kD SGs as a reference. These replicas had the worst correlation coefficients (see also Additional file 4: Table S4). (b) Comparisons in 10k1 EKCs. The comparisons with the replica b, especially between the replicas a and b, had broader scatter than one of replicas a vs. c and the 07kD replicas. The 10k1b is the only outlier in the replicas. Heat maps and the hierarchical clustering of significantly expressed cytokeratins (c) and glycolysis-gluconeogenesis (d) genes including the 10k1b replica. Some genes of the 10k1b were overrepresented, although the trends were similar. [file 12864_2015_1671_MOESM6_ESM.pdf]

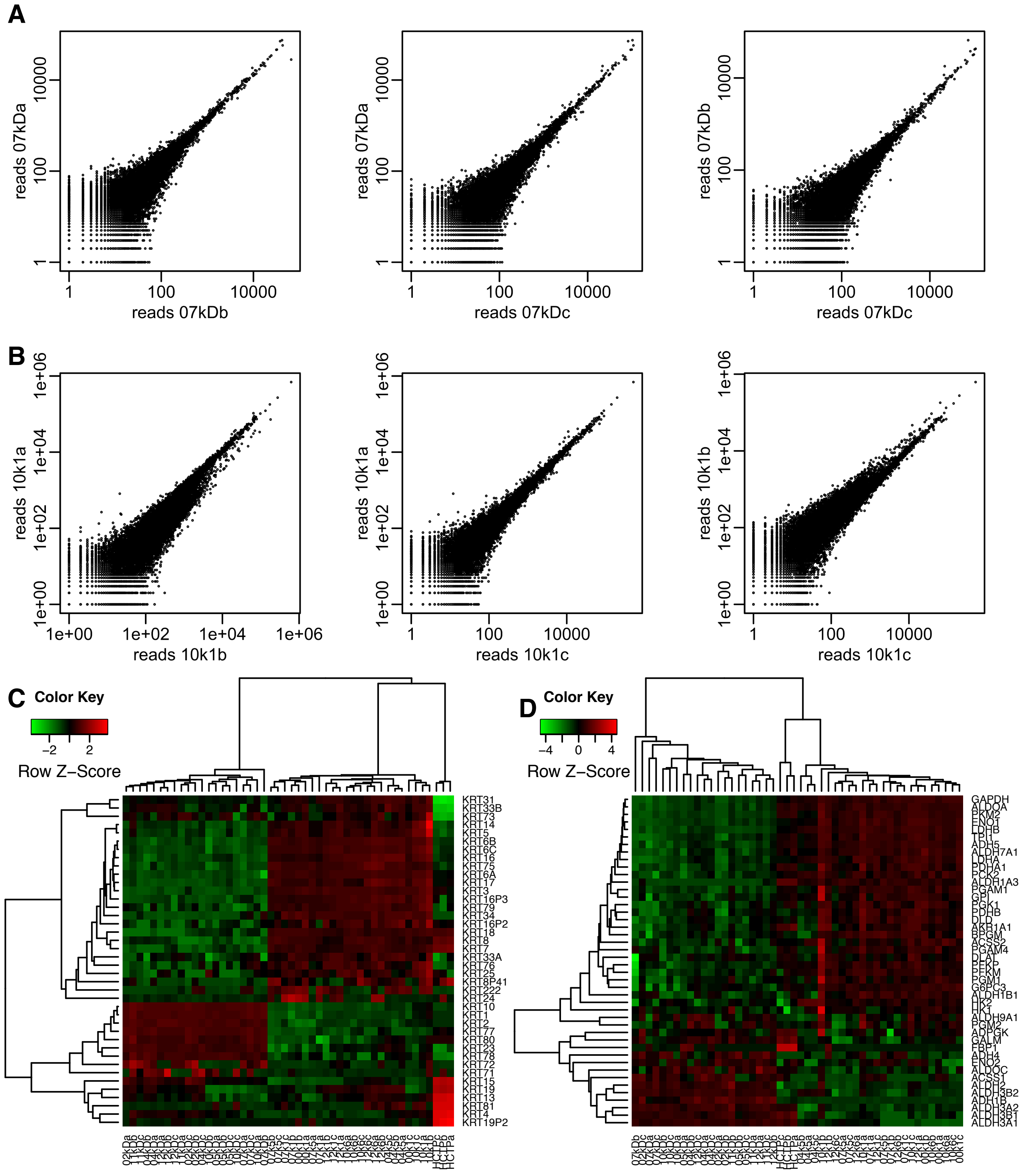
A
reads 07kDa
reads 07kDb
reads 07kDa
reads 07kDc
reads 07kDb
reads 07kDc
B
reads 10k1a
reads 10k1b
reads 10k1a
reads 10k1c
reads 10k1b
reads 10k1c
C
Color Key
Row Z−Score
KRT31
KRT33B
KRT73
KRT14
KRT5
KRT6B
KRT6C
KRT16
KRT75
KRT6A
KRT17
KRT3
KRT16P3
KRT79
KRT34
KRT16P2
KRT18
KRT8
KRT7
KRT33A
KRT76
KRT25
KRT8P41
KRT222
KRT24
KRT10
KRT1
KRT2
KRT77
KRT80
KRT23
KRT78
KRT71
KRT72
KRT15
KRT19
KRT13
KRT81
KRT4
KRT19P2
D
Color Key
Row Z−Score
GAPDH
ALDOA

Figure S2
